# Case report: Paravalvular regurgitation post transcatheter aortic valve replacement: When in doubt choose cardiac magnetic resonance

**DOI:** 10.3389/fcvm.2022.925120

**Published:** 2022-08-23

**Authors:** Michael B. Hadley, Francesca Romana Prandi, Francesco Barillà, Samin Sharma, Annapoorna Kini, Stamatios Lerakis

**Affiliations:** ^1^Zena and Michael A. Wiener Cardiovascular Institute, Icahn School of Medicine at Mount Sinai, New York, NY, United States; ^2^Division of Cardiology, Department of Systems Medicine, University of Rome “Tor Vergata”, Rome, Italy

**Keywords:** transcatheter aortic valve replacement (TAVR), paravalvular leak (PVL), cardiac magnetic resonance (CMR), multimodality imaging, case report, structural heart disease

## Abstract

Paravalvular leak (PVL) is a common complication following transcatheter aortic valve replacement (TAVR). Significant PVL is associated with adverse prognosis, but may be challenging to assess accurately. We report the case of an 81-year-old man with shortness of breath 5 months post TAVR. Echocardiography classified PVL as either moderate or severe depending on the parameter utilized, while angiography found only mild PVL. Cardiac magnetic resonance allowed an exact quantification of regurgitant flow volume, classified as clinically and hemodynamically significant. This case highlights the role of multimodality imaging assessment including cardiac magnetic resonance for a more accurate assessment of PVL severity, especially when other imaging modalities show discordant results.

## Introduction

Paravalvular leak (PVL) is a potential complication following transcatheter aortic valve replacement (TAVR), due to incomplete sealing of the aortic annulus by the bioprosthesis. Significant PVL is associated with adverse prognosis (1), may be challenging to assess accurately, and should be treated appropriately when diagnosed (2, 3).

## Case report

We present a case of an 81-year-old man that presented with progressively worsening shortness of breath 5 months post transfemoral TAVR with a 29 mm Medtronic Evolut PRO + valve. His medical history included hypertension, dyslipidemia, diabetes mellitus, chronic kidney disease, iron deficiency anemia, paroxysmal atrial fibrillation on anticoagulation, coronary artery disease treated with percutaneous coronary angioplasty and benign prostatic hyperplasia. His family history did not include any significant disease. Physical examination documented regular cardiac rhythm with 3/6 end-diastolic murmur and 3/6 mid-systolic murmur, with lungs clear to auscultation bilaterally.

## Multiparametric diagnostic assessment

**Figure 1:**
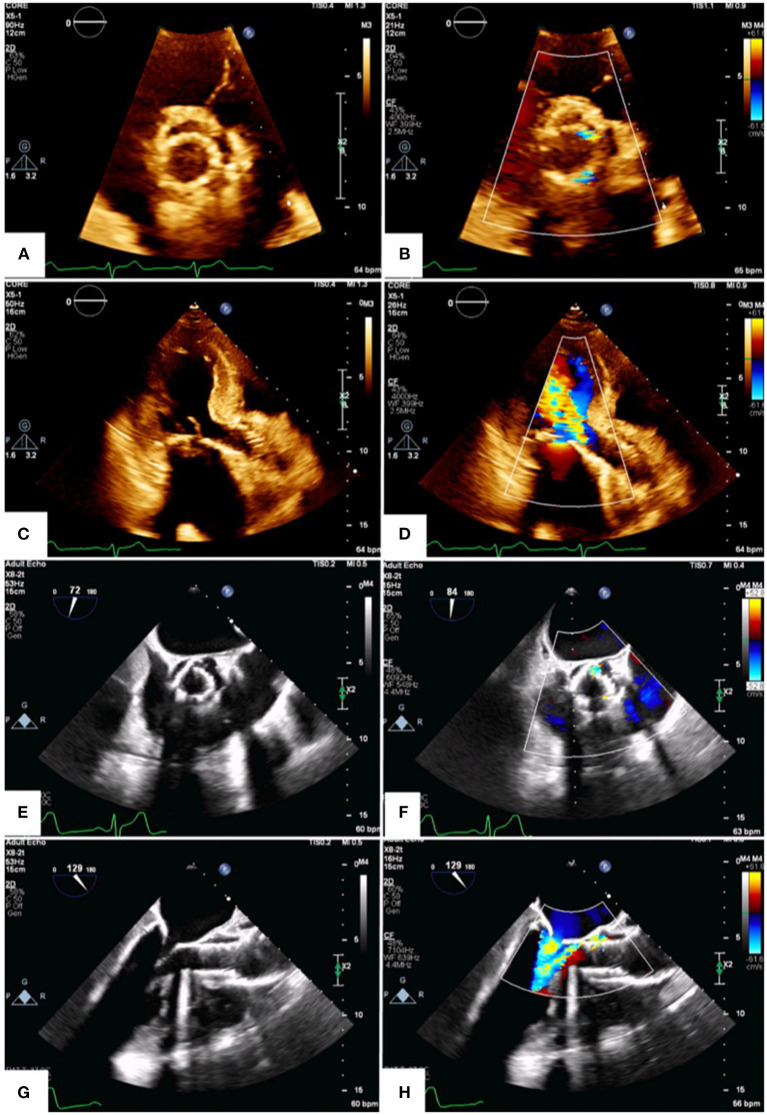
Transthoracic and transesophageal echocardiography. Transthoracic **(A–D)** and transesophageal **(E–H)** echocardiograms demonstrate multiple PVL jets on short-axis view **(B,F**; Supplementary Videos 1, 2). The largest jet is in the 1 o'clock position and is well-visualized on long-axis views **(D,H)**; (Supplementary Videos 3, 4).

**Figure 2:**
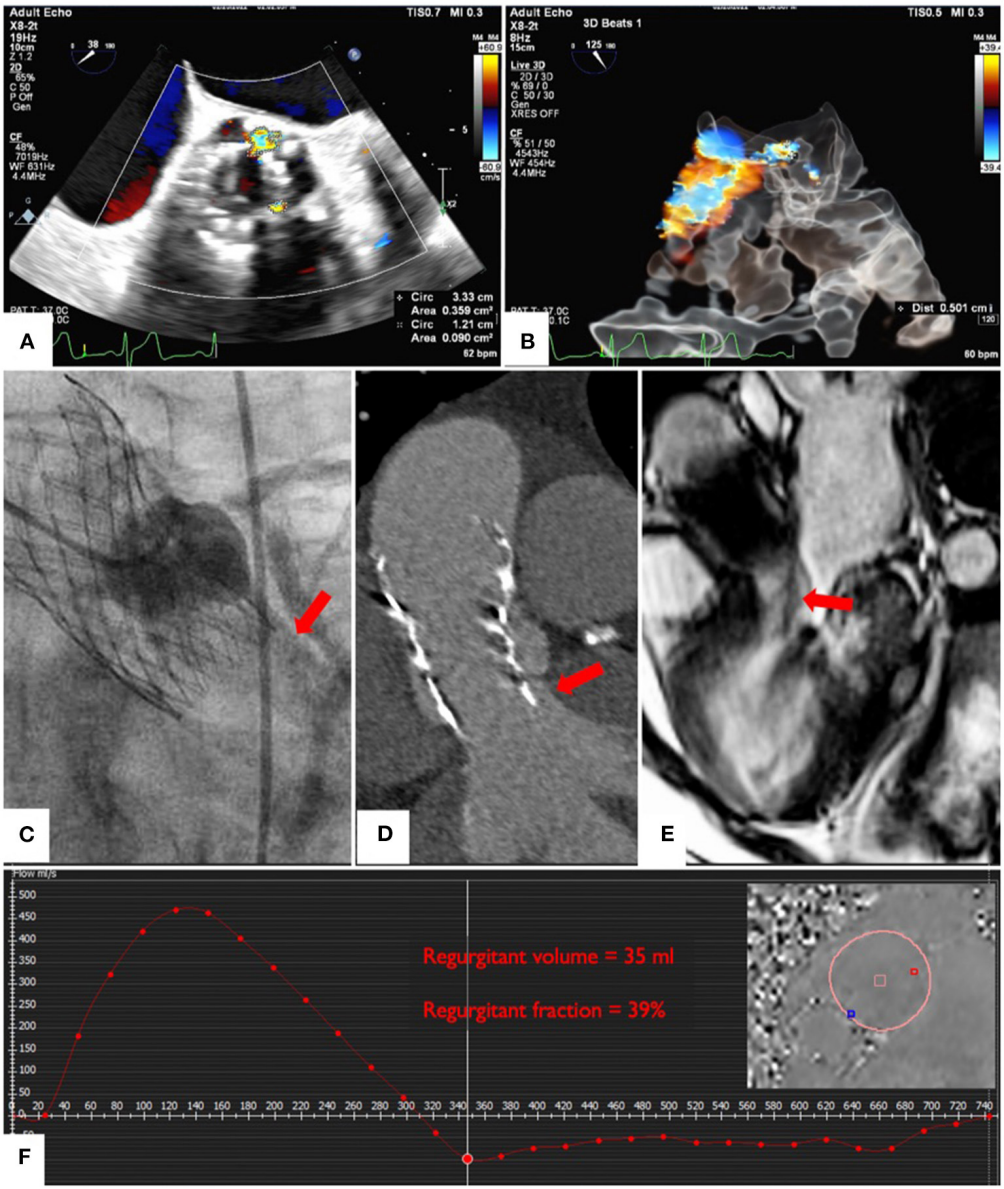
Multiparametric assessment of PVL severity. Echocardiographic quantification of jet size by planimetry **(A)** and by VC using glass image processing **(B)**; (Supplementary Video 5). Angiographic assessment of PVL **(C)**. Computed tomography showing prosthesis under-expansion **(D)**. CMR of the largest PVL jet **(E)** and quantification of the total regurgitant volume and fraction **(F)**; (Supplementary Video 6).

Echocardiography demonstrated normal biventricular function (left ventricular ejection fraction 68%), severe mitral stenosis, multiple jets of prosthetic aortic PVL classified as either moderate or severe depending on the parameter^2^ (Figure 1; Supplementary Videos 1–4) and otherwise normal prosthetic valve function (peak velocity 2.16 m/sec, mean transvalvular gradient 9 mmHg, Effective Orifice Area 1.8 cm2, Effective Orifice Area Indexed 0.98 cm2/m2). The largest PVL jet vena contracta (VC) area measured 0.36 cm^2^ (Figure 2A) with a large flow convergence, suggestive of severe PVL. However, the VC width measured 0.5 cm on glass image processing (Figure 2B; Supplementary Video 5) and the circumferential extent of the PVL was <30%, suggesting moderate PVL. Conversely, angiography found only 1+ PVL (Figure 2C). Computed tomography (CT) demonstrated that the cause of the PVL was the under-expansion of the prosthesis (Figure 2D).

Cardiac magnetic resonance (CMR) phase contrast imaging allowed exact quantification of flow through the proximal aorta (Figures 2E,F; Supplementary Video 6) bypassing the need to assess multiple jets separately (2–4). On CMR, the regurgitant fraction (RF) was 39% with regurgitant volume of 35 ml, most consistent with clinically and hemodynamically significant PVL.

Based on CMR evaluation and following Heart Team discussion, the patient underwent surgical explant of TAVR, re-do aortic valve replacement (AVR) with a 23 mm St. Jude Medical Epic Supra porcine tissue heart valve, mitral valve replacement (MVR) with a 25 mm St. Jude Medical Epic tissue heart valve and left atrial appendage ligation, followed by clinical improvement and no residual aortic regurgitation on follow-up echocardiograms.

## Discussion

Echocardiography is the first-line modality to assess PVL, but may be limited by a subjective assessment of multiple, eccentric jets with irregular orifices (4). Both transthoracic and transesophageal echocardiography may also present attenuation due to native calcification and implanted prosthetic material (5).

CMR is highly reproducible and offers an accurate, quantitative approach that bypasses the need to identify and characterize individual jets (2–6); it is particularly useful in patients with difficult acoustic windows. In symptomatic post-TAVR patients, CMR commonly reclassifies PVL grade at least one grade lower compared to semi-quantitative echocardiography, and at least one grade higher compared to qualitative echocardiography (5). CMR is technically feasible for PVL assessment in the currently approved balloon-expandable and self-expandable valves.

This approach has applications beyond TAVR. Compared with transesophageal echocardiography (TEE), CMR appears to have higher sensitivity to detect significant periprosthetic regurgitation after both surgical AVR or MVR (7, 8). Semiquantitative TEE underestimates a considerable number of AVR or MVR PVL (7). 4D flow CMR has also been used in a patient to detect a PVL after pulmonary valve replacement, and it was helpful to accurately quantify an effective regurgitant volume for decision making (9).

Accurate assessment of PVL severity is important, given implications for prognosis and management. CMR derived PVL quantification was shown to provide prognostic value superior to both qualitative and semi-quantitative echocardiography (5).

A higher RF as determined by CMR post-TAVR has been independently associated with late cumulative all-cause mortality [hazard ratio (HR) 1.18 for each 5% increase in RF; 95% confidence interval (CI): 1.08–1.30; *p* < 0.001], the combined endpoint of late cumulative all-cause mortality and heart failure (HF) rehospitalization (HR 1.19 for each increase of 5%; 95% CI: 1.15–1.23; *p* < 0.001) and the combined endpoint of late cardiovascular mortality, HF rehospitalization or reintervention in the transcatheter valve (HR 1.25 for each increase of 5%; 95% CI: 1.17–1.34; p < 0.001) (6). Furthermore, CMR-quantified AR performed at a median of 40 days post-TAVR had a greater association with post-TAVR clinical events compared with early (median of 6 days post-TAVR) echocardiography (*p* < 0.01). This demonstrates the important additional value of CMR-quantified AR in predicting clinical events beyond that of echocardiography-quantified AR (6). A RF ≥30% was associated with increased all-cause mortality (*p* < 0.001) and mortality and heart failure rehospitalization (*p* < 0.001) at 2-year follow-up (6).

Therefore, CMR may help to identify patients with severe PVL that should be considered for corrective intervention; it is particularly useful in patients with mild-to-moderate PVL on transthoracic echocardiography but who have signs/symptoms of heart failure, and those with moderate-to-severe PVL on echocardiography (10).

Overall, multimodality imaging assessment including CMR can provide a more accurate assessment of PVL severity, especially when a therapeutic intervention is under consideration and other imaging modalities show discordant results.

## Data availability statement

The original contributions presented in the study are included in the article/Supplementary materials, further inquiries can be directed to the corresponding author.

## Ethics statement

Written informed consent was obtained from the relevant individual for the publication of any potentially identifiable images or data included in this article.

## Author contributions

Conception and design: SL. Drafting of the manuscript: MH, FP, and SL. Manuscript revision: MH, FP, FB, SL, SS, and AK. Final approval of the manuscript submitted: SL, SS, and AK. All authors have read and approved the manuscript.

## Conflict of interest

The authors declare that the research was conducted in the absence of any commercial or financial relationships that could be construed as a potential conflict of interest.

## Publisher's note

All claims expressed in this article are solely those of the authors and do not necessarily represent those of their affiliated organizations, or those of the publisher, the editors and the reviewers. Any product that may be evaluated in this article, or claim that may be made by its manufacturer, is not guaranteed or endorsed by the publisher.
